# The Sign of the Spinal Muscles in Pleurisy

**Published:** 1911-04-08

**Authors:** 


### The Sign of the Spinal Muscles in Pleurisy.

Dr. Felix Ramond calls attention in the *Progrès Médical* to a new diagnostic sign which he has met with in cases of pleurisy and which he names the sign of the spinal muscles. On examining the back of a normal individual in the standing position, the outline of the two superficial spinal muscles, the ileo-costalis externally and the longissimus dorsi internally, can be readily made out. Palpation of the lumbar region shows these muscles to be in a state of tension, and their mass varies in thickness according as one or both of them enters into action. In patients with pleurisy, however, the two muscles are in a state of permanent reflex contraction on the affected side, and from this several phenomena result which, taken together, constitute the sign of the spinal muscles. On first inspection the muscular mass in the lumbar region appears larger and more projecting on the side of the pleurisy. On palpation it gives a sensation of hardness and resistance to pressure quite different from that of the mass on the sound side. If the upper attachments of the middle portion of the longissimus dorsi be hit with a percussion hammer at the level of the sixth or seventh dorsal vertebra two or three fingers-breadth from the middle line, while at the same time the muscular mass is palpated, the muscles will be found to vibrate under the blow. This sign, in the absence of others, is not diagnostic of a pleural effusion, since it is met with in patients whose muscular reflexes are exaggerated, such as cachectics, alcoholics and neurasthenics. The other two signs are, however, pathognomonic. They may all be present in the one case, or may appear separately. Objective enlargement is, however, constant.

If the other signs appear to be absent they can be elicited by making the patient bend from side to side. Normally the muscles on the flexed side are completely relaxed, while those on the opposite side are more tense and harder. If pleurisy is present, *e.g.*, on the right side, flexion to the right will not give a sensation of complete relaxation of the muscles, whereas flexion to the left makes them of extraordinary hardness, especially if their superior attachments be struck with the hammer. The author has noticed in a few cases that the sign would seem to have spread somewhat to the sound side. In these he has found that a large effusion was present corresponding to the triangle called Groceo's. Out of seven cases of this sort seen by the author he has in three succeeded in removing a small quantity of fluid from the inferior cul-de-sac of this supposedly normal pleura.

The duration of the sign exceeds that of the pleurisy by three or four weeks, and thus serves to establish a retrospective diagnosis. In three cases it has been noticed as late as six months, nine months, and three years after. Its persistence is of bad diagnostic import and indicates that the inflammatory process has continued after the disappearance of the effusion, and that cure is more apparent than real. The sign is constant in primary pleurisy, and the author has found it in 94 per cent. of cases of pleurisy due to pulmonary tuberculosis. It was met with in all three cases of pleurisy from Bright's disease seen by the author, but only in five out of eight cases of pleurisy from heart disease. In seven cases of suppurative pleurisy it was present four times, and the curious observation was made that the sign tended to become negative in proportion as the purulent inflammation of the pleura increased. It would seem to be of certain diagnostic importance with regard to inflammatory pleural effusions, for it was not present in three cases of pure pneumothorax without reaction of the serous membranes, nor in three out of five cases of pneumonia, its presence in the other two being no doubt due to an accompanying pleural inflammation. The author thinks the sign of importance in that all other signs of pleurisy are inconstant and indefinite with the exception of dulness. He acknowledges that it is of secondary value, but in view of its constancy and specificity he maintains that it is worthy of attention in doubtful cases, which are not so rare as might be imagined. At any rate, the sign is an interesting one which demands to be known widely so that its value may be thoroughly tested.

